# Capital Punishment—The Kemmler Case

**Published:** 1890-08

**Authors:** 


### CAPITAL PUNISHMENT:—THE KEMMLER CASE.

All the world knows now the details of the execution by electricity of Wm. Kemmler in Albany, New York, on the 6th inst. it is notorious,—being the first judicial murder ever perpetrated by means of the electric current.

If the death of the criminal was the end sought, the execution was a success; for he was killed, very dead, no doubt, at the first contact; the opinion of "expert" Spitza to the contrary, notwithstanding. If a less revolting, less horrible and disgusting method of putting a man to death than by hanging was the desideratum sought in the electro killing, it was in our judgment, a lamentable failure. It was simply horrible, even in itself, as detailed in the daily press; but when are added to the account the incidents connected with the killing, the scene becomes simply revolting,—to say nothing of the refinement of cruelty in keeping the wretch in the dreadful suspense and uncertainity as to when he was to be killed,—the leaving of the hour to the warden's selection at any time between the limits of a week. To—

> "Sit in solemn silence in a dull dark dock
> In a pestilential prison with a life long-lock
> Awaiting the sensation of a short sharp shock,"

as Sullivan and Gibert put it, in Mikado, is distressing enough; but to know that that "sensation," which shall terminate his life, is to occur at some time between Sunday morning and Saturday night, to realize that he may be called to death at any moment, night or day—is, as said, simply the very essence and refinement of torture. All things combined to render this execution—if the bungling murder of that unfortunate wretch could be dignified with the name, the most horrible in the annals of crime. First the cool, unconcerned manner of the condemned, which was neither stoicism nor bravado, but which seemed to be a dulled intellect rendered, in mercy, incapable of comprehending the fearful situation, notwithstanding that the certainty of death, without the shadow of a hope of escape or reprieve, must have occupied his mind; the deliberate fixation in the dreadful chair, the *sang froid* with which the culprit assisted; but after contact and the passage of the 1000 volts through his frame for seventeen seconds, which must have killed him instantly, came the climax of the horrors. As might have been anticipated, there was muscular contraction of the walls of the thorax, and with the escape of the residual air of the lungs, as we take it to have been, there was froth oozing from the mouth of the corpse. Instantly, Spitza lost his head and screamed "turn on the current, this man is not dead!" Whereupon, after a delay of minutes, which to the frightened lookers-on seemed months, the lever was turned, the circuit completed, and the current passed through the corpse some three minutes more, and until the skin at points of contact with the electrodes was burned, emitting the dreadful stench of burning flesh! Could anything have been more revolting? And, in perfectly cold blood! The whole party must have felt like a lot of murderers, who, having sand-bagged an inoffensive (to them) victim, and seeing signs of returning consciousness, as they ignorantly supposed, called out "hit him again, he isn't dead yet!"

There is no justification of murder. A homicide in hot blood, the killing of a human being when one is laboring under some strong emotion, some sense of great wrong, is sometimes pardonable; but to deliberately put to death a human being with the utmost careful forethought as to every detail, is something of which it would seem the human heart would be incapable.

If murder be wrong, how can two murders be right? What right has the State to commit murder more than an individual? for every execution is a murder, if not with "malice" certainly with intention, deliberately "aforethought;" and the judicial murder is always, for that reason, the most atrocious of the two.

Capital punishment is a remnant of our barbarism,—almost the sole remaining one, and should be abolished. Every consideration of justice, morality, humanity and civilization demand it. It is to be condemned on every head. Morally, (?) it is a failure, if the excuse be that it is intended to diminish crime, to awe evil doers and restrain them from violence; for statistics show that in those States where capital punishment is inflicted for murder, crime is not on the decrease, rather, on the increase, if we are not mistaken, and instances have been known where an execution has seemed to act as a suggestion to crime. A case of brutal murder was committed in Mississippi, by a negro who had recently witnessed the hanging of another negro of his acquaintance.

The many chances to escape punishment, too, offered by the "law's delays," conspire to render evil-disposed persons very indifferent to the death sentence.

Economically considered, every execution of an adult male robs the State of an equivalent of about $1000 a year. Statisticians place each man's money value, we believe, at about that figure. On the score of economy, then, leaving every other consideration out of sight, it would be far better to imprison the murderer for life, and put him to work for the benefit of that world which he has outraged and forfeited.

We agree with our able contemporary of the *New York Medical Record* that the time is now ripe for a crusade looking to the abolishment of capital punishment. In speaking of the Kemmler murder, the *Record* says: "The death chair in which Kemmler expiated his crime may yet be the pulpit from which will be preached the doctrine of abolition of capital punishment." God grant it, and may it soon begin. There is no justification for either hanging, shooting, or otherwise deliberately putting to death one who has offended the laws of the State. Under the old Mosaic dispensation, "an eye for an eye, a tooth for a tooth and a life for a life," was a law rendered necessary perhaps by the then want of civilization; but in this nineteenth century—a time of advanced civilization, of civil and religious liberty, of great humanity—capital punishment is a blot and stain on the escutcheon of any civilized government.

But, as to the question whether electrothanasia is preferable to death by the rope, since the death penalty must be inflicted, it strikes us that, if properly managed, electro-execution could be made to rob the occasion of most that is revolting; but a repetition of the Albany cruelty—a farce but for its horrors—we believe would arouse the indignation of the people to the point of demanding the repeal of the law.

